# Can We Predict Psychosis Outside the Clinical High-Risk State? A Systematic Review of Non-Psychotic Risk Syndromes for Mental Disorders

**DOI:** 10.1093/schbul/sbx173

**Published:** 2018-02-09

**Authors:** Tae Young Lee, Junhee Lee, Minah Kim, Eugenie Choe, Jun Soo Kwon

**Affiliations:** 1 Department of Psychiatry, Seoul National University College of Medicine, Seoul, Republic of Korea; 2 Department of Brain and Cognitive Sciences, Seoul National University College of Natural Sciences, Seoul, Republic of Korea

## Abstract

Recent evidence has suggested that psychosis could develop not only in people at clinical high risk for psychosis (CHR-P) but also in those with clinical risk syndromes for emergent nonpsychotic mental disorders. The proportion of people with these clinical risk syndromes who will develop psychosis rather than to other nonpsychotic mental disorders is undetermined. Electronic databases were searched for studies reporting on clinical risk syndromes for the development of emergent nonpsychotic mental disorders. Incidence of emerging psychotic and nonpsychotic mental disorders defined on the ICD or DSM. Of a total of 9 studies relating to 3006 nonpsychotic at-risk individuals were included. Within prospective studies (*n* = 4, sample = 1051), the pooled incidence of new psychotic disorders across these clinical risk syndromes was of 12.9 per 1000 person-years (95% CI: 4.3 to 38.6) and that of nonpsychotic disorders (*n* = 3, sample = 538) was of 43.5 per 1000 person-years (95% CI: 30.9 to 61.3). Psychotic disorders may emerge outside the CHR-P paradigm, from clinical risk syndromes for incident nonpsychotic disorders, albeit at lower rates than in the CHR-P group. The clinical risk syndromes for emerging nonpsychotic disorders may exhibit a pluripotential risk of developing several types of mental disorders compared with CHR-P. If substantiated by future research, the current findings suggest that it may be useful to move beyond the current strategy of identifying individuals meeting CHR-P criteria only.

## Introduction

In clinical medicine, a true prodrome refers to the early symptoms and signs that inevitably precede the acute clinical phase of an illness.^1^ The term prodrome originates from the Greek word “prodromos,” meaning “precursor”; by definition, it is usually a retrospective concept. In contrast, the term clinical high risk for Psychosis (CHR-P) state describes a condition with particular clinical features that lead to an elevated risk of developing the illness.^2^ Thus, this term is prospective, and an individual in a CHR-P state may or may not subsequently progress to the illness (see supplementary material [eIntroduction] for further details).^3^

Meta-analytic evidence indicates that approximately 22% of CHR-P criteria develop psychosis within 3 years (see eFigure 4 published in ref.^4^). This meta-analysis confirmed that the transition rates for psychosis had declined annually, although not in all CHR-P sites,^5^ in contrast to the results of earlier studies.^6,7^ In addition to the observation that approximately 78% of individuals at CHR-P do not go on to develop full psychosis, one-third of them only were reported to remit from the initial CHR-P status in the following years.^8,9^ Several possible explanations regarding this phenomenon have been proposed, such as more effective early intervention, lead-time bias and the identification of false positives who were never at-risk of psychosis.^10–12^ The latter point is likely due to heterogeneous recruitment strategies and dilution of enrichment before the CHR-P assessment, an issue that has been extensively addressed in recent publications.^12–14^ More importantly, 2 independent studies confirmed that the CHR-P criteria have proven to be accurate for predicting the onset of psychosis but not of nonpsychotic disorders.^15,16^ This finding contradicts earlier assumptions that CHR-P is pluripotential in term of diagnostic outcomes (eg, with an “At-Risk Mental State, ARMS” predicting several incident mental disorders). At the current stage of knowledge, what is not clear is whether individuals with other clinical risk syndromes beyond the CHR-P may also be at-risk of developing psychosis.

Mood disorders, including bipolar disorder, are commonly seen in childhood and adolescence, and this period significantly overlaps with the CHR-P stage of psychosis.^17,18^ It is also well known that obsessive-compulsive disorder shares some phenotypical aspects with psychosis and is often observed at the CHR-P phase of psychosis.^19^ Similarly, in the pre-onset state of depression, 3% of people with subthreshold depression who did not meet the diagnostic criteria for major depressive disorders developed schizophrenia in 3–4 years of follow-up.^20^ Moreover, a recent meta-analysis confirmed that 1.56% of help-seeking individuals who did not meet the CHR-P criteria developed psychosis after 38 months.^21^ This result suggests that psychosis can develop not only in CHR-P individuals but also in those with clinical risk syndromes for nonpsychotic disorder or nonpsychotic mental disorders. However, defining the clinical risk syndromes for nonpsychotic mental disorders includes various considerations due to trans-diagnostic complexity, in particular when employing subthreshold symptoms.^22^ For example, when a person has subthreshold symptoms that do not meet the diagnostic criteria for depressive/anxiety disorders, it is not easy to differentiate whether this condition could be defined as a clinical risk state for nonpsychotic disorders or a just mood fluctuation in normal ranges, because it is difficult to assume that these disorders also have life-long trajectories like schizophrenia. Similarly, if a patient already diagnosed with a mental illness such as dysthymic disorder or attention-deficit/hyperactivity disorder (ADHD), and these later develop into a major depressive disorder or bipolar disorder, it is unclear whether the former diagnoses are reasonable to also define a risk state of a subsequent mental disorder at that time. In the lack of a coherent and validated conceptual framework to define clinical risk syndromes for the nonpsychotic mental disorders, we have adopted a pragmatic approach and operationalized them as follows: (1) individuals with subthreshold symptoms other than CHR-P symptoms who do not meet the diagnostic criteria for an established ICD or DSM nonpsychotic mental disorders and/or (2) individuals receiving an established ICD or DSM diagnosis of nonpsychotic mental disorder (other than CHR-P) but who later develop into more severe conditions.

This area of research is relatively underexplored, and we currently do not have sufficient understanding of the extent to which emergent psychotic disorders vs nonpsychotic disorders may originate from clinical risk syndromes other than the CHR-P, and what factors may constitute significant predictors. The main aim of the current systematic review is to report on the risk of developing emergent psychotic-disorders from clinical syndromes other than the CHR-P. Extending our knowledge of the risk of developing these outcomes would allow us to inform the next generation of research studies in this area.

## Methods

### Search Strategy

A systematic search strategy was employed to identify relevant literature. Two independent researchers (J.L. and E.C.) conducted a 2-step literature search. First, a literature search using PubMed, EMBASE, and the Cochrane Library was performed to identify relevant studies from database inception to November 2016. The following keywords, including their synonyms and combinations, were used as search terms: “depressi*”, “affective”, “mood”, “bipolar”, “manic”, “mania”, “anxiety”, “panic”, “social phobi*”, “obsessi*”, “attenuated”, “prodrom*”, “incipient”, “early”, “at-risk”, “high-risk”, “prevent*”, “subthreshold”, “adolescen*”, “predict*”, “preclinical”, “longitudinal”, “outcome”, “trajectory”, “course”, “transition”, “onset”, “conversion”, “convert*”, “develop*”, “incident”, “incidence”, “psychotic”, “psychosis”, and “schizophren*”. In the second step, the reference lists of the identified reviews and studies were manually checked to identify additional relevant publications. The eligible articles were selected according to the Meta-analyses and Systematic Reviews of Observational Studies (MOOSE) checklist (supplementary material [eTable 1]).^23^

### Selection Criteria

**Fig. 1.**
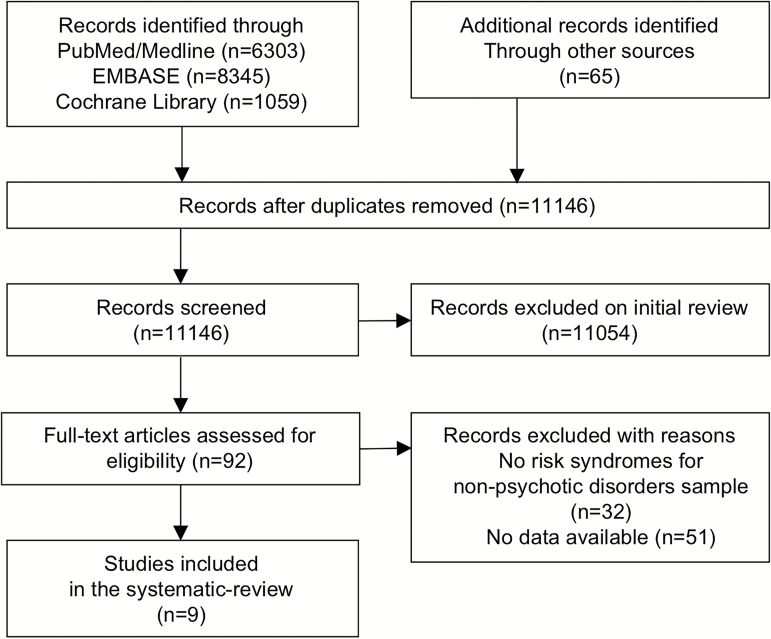
PRISMA flow-chart.

Studies were included if they met the following criteria: (1) original articles, written in English; (2) inclusion of clinical risk syndromes for emergent nonpsychotic mental disorders; (3) studies reporting data enabling calculations of the incidence of psychotic or nonpsychotic disorders outcomes using established international diagnostic manuals (ICD or DSM). In cases of sample overlap, the study with the larger sample size was included. We define the criteria of clinical risk syndromes for emergent nonpsychotic mental disorders as follows: (1) individuals with subthreshold symptoms other than CHR-P symptoms that do not meet the diagnostic criteria for established nonpsychotic mental disorders (eg, those at-risk for bipolar disorders) and/or (2) individuals receiving an established diagnosis of nonpsychotic mental disorder (other than CHR-P) but who later develop into more severe conditions (eg, from Dysthymia to Bipolar Disorders). Of relevance, since individuals that are assessed with CHR-P instruments but who are not meeting CHR-P criteria may be meeting the above inclusion criteria, they were also included in the current review as an additional exploratory group. Exclusion criteria were (1) studies employing general population samples not stratified across at-risk sub-groups that reflect specific subthresholded symptoms; (2) lack of outcome data (ie, onset of emerging psychotic disorders). The literature search was conducted according to the PRISMA guidelines (figure 1).^24^

### Statistical Analysis

Although this is primarily a systematic review, we also performed some exploratory quantitative analyses within prospective studies. First, the incidence of psychotic and nonpsychotic disorders in each clinical risk syndrome was calculated by dividing the number of incident diseases by the total number of person-years of observation. If the exact mean duration was unavailable, the mean of the minimum and maximum follow-up duration was used. Second, we pooled the studies using the Poisson distribution, to calculate 95% CIs for the incidence rates of emerging psychotic and nonpsychotic disorders across the whole group. Random-effects models were then applied using the log incidence rates and corresponding standard errors. All statistical analyses were performed with Stata v14.

## Results

The initial literature search identified 92 eligible articles from 11146 articles (PRISMA Flow-chart, see figure 1). Nine articles were meeting the inclusion criteria. A full list of included studies is presented in table 1. The majority of the studies (*n* = 5) focused on bipolar risk; other studies of clinical risk syndromes for emergent nonpsychotic disorders investigated depressive risk (*n* = 2), OCD (*n* = 1), and panic risk (*n* = 1). The database included a total of 3006 nonpsychotic at-risk subjects. However, there were only 4 prospective studies comprising 1051 participant, and 5 retrospective studies comprising 1955 participants. Most of the retrospective studies reported psychotic outcomes simply as a part of the general diagnostic outcome and did not examine baseline differences in the characteristics of at-risk patients who developed emergent psychotic and nonpsychotic outcomes. Finally, as mentioned in the methods we additionally included a comparative group of 2519 subjects (*n* = 11) assessed for CHR-P risk but not meeting intake criteria (table 2).

**Table 1.** Eligible Studies Including Risk Syndromes for Nonpsychotic Disorders Reporting on Psychotic and Nonpsychotic Disorders

Studies	Baseline Assessments	Prospective Design	Sample	Follow-up Duration (y)	Outcome	Transition to Psychosis^c^	Transition to Nonpsychosis^c^
Depression risk							
Akiskal et al^20^	WUC	Y	Subthreshold depression (*n* = 100)	3~4	Schizophrenia^a^ (*n* = 2) Schizoaffective disorder^a^ (*n* = 1) Unipolar disorder^a^ (*n* = 22)	8.6	62.9
Weiser et al^25^	ICD-10	Y	Community or nationwide sample (*n* = 275705) Dysthymia (*n* = 513)	7.9 ± 1.8	Psychosis^b^ (*n* = 10) Schizophrenia^b^ (*n* = 4)	3.5	N/A
Bipolar risk							
Alloy et al^54^	GBI exp-SADS-L exp-SADS-C	Y	Community sample (*n* = 20500) Bipolar spectrum disorder (*n* = 201)	4.54 ± 2.74	Bipolar I disorder with psychosis^a^ (*n* = 22) Bipolar I disorder without psychosis^a^ (*n* = 9) Bipolar II disorder^a^ (*n* = 24)	24.1	34.0
Conus et al^27^	SCID-I IMPQ	*N*	Sample with prodromal symptoms (*n* = 22)	0.4 ± 0.31	Bipolar I disorder with psychosis^a^ (*n* = 15) Schizoaffective disorder^a^ (*n* = 7)	N/A	N/A
Correll et al^18^	BPSS-R	*N*	Sample with prodromal symptoms (*n* = 52)	1.8 ± 1.7	Bipolar I disorder with psychosis^a^ (*n* = 34) Bipolar I disorder without psychosis^a^ (*n* = 52)	N/A	N/A
Faedda et al^65^	SCID K-SADS	*N*	Sample with prodromal symptoms (*n* = 82)	7.8 ± 3.8	Bipolar disorder with psychosis^a^ (*n* = 26) Bipolar disorder without psychosis^a^ (*n* = 56)	N/A	N/A
Faravelli et al^66^	MINI FPI	*N*	Community sample (*n* = 2363) Subthreshold bipolar disorder (*n* = 110)	N/A	Psychotic disorder^a^ (*n* = 1) Phobia^a^ (*n* = 19), Obsessive- compulsive disorder^a^ (*n* = 19), Generalized Anxiety Disorder^a^ (*n* = 43), Others^a^ (*n* = 7)	N/A	N/A
Obsessive-compulsive risk							
Van Dael et al^26^	CIDI CAN	Y	Community sample (*n* = 7076) Subthreshold obsessive- compulsive risk (*n* = 237)	3	Psychotic disorder^a^ (*n* = 24) Obsessive- compulsive disorder^a^ (*n* = 29)	33.8	40.8
Panic risk							
Goodwin et al^67^	DIS	*N*	Community sample (*n* = 20291) Subthreshold panic disorder (*n* = 1689)	N/A	Schizophrenia^a^ (OR=2.545, 95%CI=2.251– 2.838) Bipolar disorder^a^ (OR=0.152, 95%CI=0.018– 0.286)	N/A	N/A

*Note*: WUC, Washington University Criteria; GBI, Revised General Behavior Inventory; exp-SADS-L, C, Expanded Schedule for Affective Disorder and Schizophrenia–Lifetime diagnostic interview, -Change; SCID-I, The Structured Clinical Interview for DSM-IV Axis I Disorders; IMPQ, The Initial Mania Prodrome Questionnaire; BPSS-R, The Bipolar Prodrome Symptom Interview and Scale-Retrospective; K-SADS, The Kiddie Schedule for Affective Disorders and Schizophrenia; MINI, The Mini International Neuropsychiatric Interview; FPI, Florence Psychiatric Interview; ICD-10, International Classification of Diseases-10; CIDI-AUTO, The Composite International Diagnostic Interview; CAN, Camberwell Assessment of Need; DIS, The Diagnostic Interview Schedule.

^a^Diagnostic and Statistical Manual of Mental Disorders (DSM).

^b^International Classification of Disease (ICD).

^c^Cases per 1000 person-years.

**Table 2.** The 3-Year Risk of Developing Psychosis Across Different Samples Considered in the Current Review (Incidence (%), 95% CI)

Incident Disorder	Sample Denominator	Incident Rates of Developing Psychosis (%)	Relative Risk to General Population
Psychosis	General Population^33^	0.05 (95% CI: .02 to .13)	1-fold
Psychosis	Individuals assessed but not meeting CHR-P criteria^34–44^	1.48 (95% CI: .66 to 2.29)	29.6-fold
Psychosis	Clinical Risk Syndromes other than CHR-P	3.87 (95% CI: 1.29 to 11.58)	77.4-fold
Psychosis	Individuals undergoing CHR-P assessments^34–44^	14.21 (95% CI: .85 to 22.74)	284.2-fold
Psychosis	Individuals meeting CHR-P criteria^34–44^	24.63 (95% CI: 21.79 to 28.42)	492.6-fold

### Incidence of Emergent Psychotic Disorders in Clinical Risk Syndromes Other Than CHR-P Longitudinal Studies

The incidence rates of emerging psychotic disorders were 4.4 per 1000 person-years (95% CI: 2.0 to 9.8) for depression risk syndrome, 24.1 per 1000 person-years (95% CI: 15.1 to 36.5) for bipolar risk syndrome, and 33.7 per 1000 person-years (95% CI: 21.6 to 50.2) for obsessive-compulsive risk syndrome. There were no longitudinal studies on panic risk syndrome. When the prospective studies were pooled together (*n* = 4, sample = 1051), the incidence of emergent psychotic disorders from clinical risk syndromes for nonpsychotic disorders was 12.9 per 1000 person-years (95% CI: 4.3 to 38.6).

The incidence rates of emergent nonpsychotic disorders were 62.9 per 1000 person-years (95% CI: 39.4 to 95.2) for depression risk syndrome, 34.0 per 1000 person-years (95% CI: 23.1 to 48.2) for bipolar risk syndrome, and 40.8 per 1000 person-years (95% CI: 27.3 to 58.6) for obsessive-compulsive risk syndrome. There were no longitudinal studies reporting on nonpsychotic outcomes from panic risk syndrome. The pooled incidence of new nonpsychotic disorders from clinical risk syndromes for nonpsychotic disorders (*n* = 3, sample = 538) was 43.5 per 1000 person-years (95% CI: 30.9 to 61.3).

The incident rates of emerging psychotic disorders in the exploratory group of individuals assessed (*n* = 11, sample = 2519) assessed but not meeting CHR-P criteria was 4.9 per 1000 person-years (95% CI = 2.2 to 7.6).

### Risk Factors for Developing Psychosis in Risk Syndromes Other Than CHR-P

Across the prospective studies, one study found an association between initial dysthymia and subsequent hospitalization for psychosis during an average follow-up period of 9 years association (HR = 4.0, 95% CI: 2.1 to 7.4).^25^ Another study found an association between baseline obsessive-compulsive symptoms and the subsequent development of psychosis during a follow-up for 2 years (OR = 6.4, 95% CI: 2.5 to 16.2).^26^ This association remained significant after adjusting for any baseline psychotic symptoms.

Across the retrospective studies, a report characterized bipolar risk syndrome through a nested subsample from a large mania cohort.^27^ Individuals at clinical risk for bipolar disorder were found to have a significantly older age of patients with first-episode mania at intake (*P* < .01). Another study in bipolar risk characterized it as a state preceding the onset of both psychotic and nonpsychotic manic episode.^18^ Psychotic mania patients had an older age of onset of the first manic episode (*P* = .01) and a lower prevalence of comorbidity with ADHD (*P* < .01) than nonpsychotic mania patients. Additionally, individuals with bipolar risk syndrome who later develop psychosis showed increased energy/goal-directed activity (*P* < .01) and higher subsyndromal psychotic symptoms such as suspiciousness (*P* < .01) compared to those who later develop mania.

## Discussion

To our knowledge, this is the first systematic review exploring whether we can theoretically predict the onset of psychosis outside the CHR-P construct. To address this point, we included available clinical risk syndromes for the development of any emergent nonpsychotic disorders and reported on the incidence of both psychotic and nonpsychotic mental disorders. On a conceptual level, we observed that besides the bipolar at-risk state, other clinical risk syndromes were poorly operationalized and the concept of a clinical high-risk state has not yet been generally introduced. Given the lack of consistent approaches, in particular in anxiety and depressive disorders, we mostly restricted our analyses to prospective studies. Within these studies, we observed a pooled incidence of emerging psychotic disorders of 12.9 per 1000 person-years (95% CI: 4.3 to 38.6), and of emerging nonpsychotic disorders of 43.5 per 1000 person-years (95% CI: 30.9 to 61.3).

**Fig. 2.**
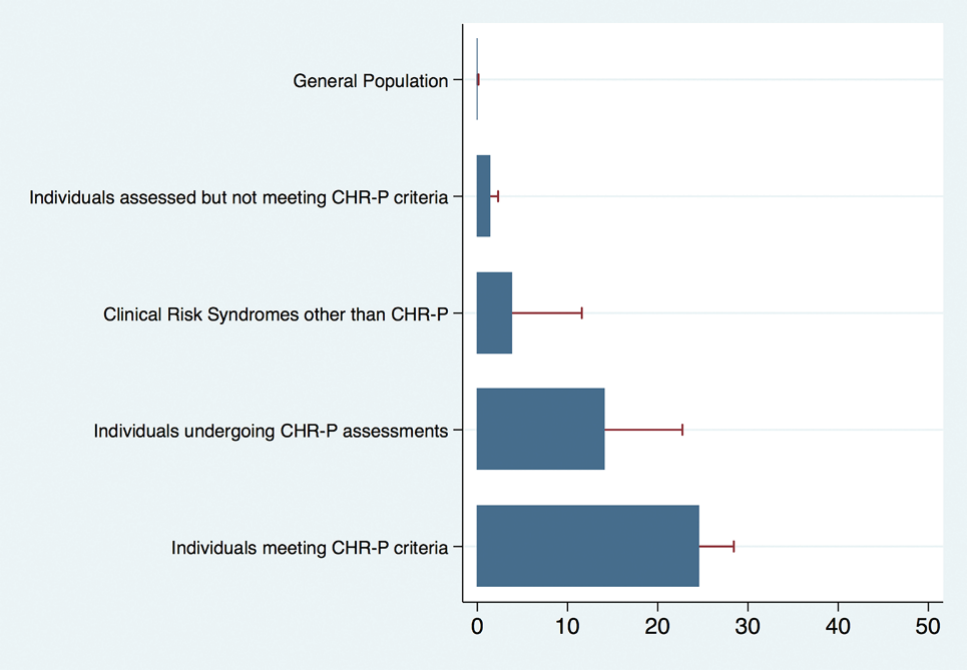
The 3-year risk of developing psychosis across different samples considered in the current review (incidence [%], 95% CI).

On a conceptual level, our review highlighted a relatively poor research in the area of clinical risk syndromes for emergent nonpsychotic mental disorders. The field was lacking clear operationalization for defining clinical risk syndromes for the development of new nonpsychotic disorders, except for bipolar at-risk states.^28,29^ Overall, in this systematic review, we found that the clinical risk syndromes for emergent nonpsychotic disorder subjects had a psychotic/ nonpsychotic incidence of 12.9 and 43.5 per 1000 person-years, respectively. For comparative purposes, these numbers could be translated into approximately 3.9% and 13.1% 3-year incidence of the psychotic/ nonpsychotic disorders, respectively. Therefore, in clinical risk syndromes for emergent nonpsychotic disorder subjects, the annualized incidence of psychotic disorder is about one-third (3.9%/ 13.1%) compared with that of nonpsychotic mental disorders. However, compared with the general population, incidence of psychotic disorders may be higher. The incidence of psychotic disorders in general population is significantly influenced by geographical, ethnical, environmental and the diagnostic criteria of psychosis, but it ranges from 0.08 to 0.54 per 1000 person-years depending on the study.^30–32^ Again for comparative purposes, this would translate in a 3-year risk of psychosis of 0.05%, which is about 1% (0.05%/ 3.9%) of the level of risk observed in clinical risk syndromes for emergent nonpsychotic disorder.^33^ These findings suggested that when studying outcomes of the clinical risk syndromes for emergent nonpsychotic disorder we should consider not only the onset of the nonpsychotic disorders but also the onset of the psychotic disorders. In a twilight zone between the development of psychotic and nonpsychotic disorders, one thing we should not disregard is the significance of the help seeking samples that underwent CHR-P assessment without meeting the intake CHR-P criteria. The recent published longitudinal study showed that these samples have a lower risk of nonpsychotic disorders (1.48% at 3-year) than that those meeting CHR-P criteria (24.63% at 3-year).^16^ On the basis of these qualitative comparisons, it is possible to roughly estimate the level of psychosis risk enrichment occurring from the general population to samples undergoing CHR-P assessment but not meeting intake criteria, those presenting with CHR-P and those presenting with clinical risk syndromes other than the CHR-P state. We present such a qualitative comparison in the figure 2. These results indicate that the samples which do not meet CHR-P criteria, the risk syndromes for nonpsychotic disorders, the samples seeking help at CHR-P services, and the CHR-P population have approximately 30-fold, 77-fold, 284-fold, and 492-fold higher risk of psychosis than that in the general population, respectively (table 2). However, the figures provided should be interpreted cautiously because they are not the result of a strict meta-analysis and because our database was rather small. Furthermore, each risk syndrome was mostly reporting on the risk of developing specific outcomes (eg, depression from subthresholded depressive symptoms) without comprehensively reporting on the risk of developing other nonpsychotic disorders. Therefore, our comparative estimates should be considered exploratory in nature and subject to further validation.

With these caveats in mind, our review has some conceptual implication. First, it questions the pluripotentiality assumption of the CHR-P state. When Johannessen and McGorry proposed the term “pluripotent risk syndrome,” the possibility that the high-risk population could develop a variety of mental illnesses, along with psychosis, was taken into consideration.^45^ The pluripotential power of CHR-P, however, is not currently supported by evidence.^2,15,16^ A recent long-term follow-up study directly demonstrated that CHR-P is not a pluripotent group for predicting the onset of new mental disorders but is specific for psychotic disorders.^15,46^ It also suggested that the nonpsychotic comorbidity of CHR-P is not the result of CHR-P but that it existed from the baseline.^16^ Meta-analytic results have confirmed that approximately 40% of psychosis risk patients already have comorbid depressive disorders at baseline.^47^ In the long-term course of CHR-P, although a large number of high-risk individuals would show nonpsychotic illnesses, most of these are carried over from baseline.^48–50^ These findings parallel what has been observed in other clinical at risk states, such as prediabetes or mild cognitive impairment, with many individuals presenting persisting disability at follow-up.^51^ Our results suggest that psychosis may emerge in risk syndromes for nonpsychotic disorders but at lower rates than in CHR-P syndrome. Furthermore, while CHR-P is specific to psychosis only, risk syndromes for nonpsychotic disorders may be more likely to have both psychosis and nonpsychotic disorders. Risk syndromes other than the CHR-P may rather hold some pluripotentiality. Although based on a small number of samples, there was a report that depressive features, which precedes the onset of mental disorders, can represent core features of a pluripotential risk state.^52,53^ However, the incidence of emerging psychosis in the depression risk population is too low to allow direct preventative approaches. In the case of bipolar risk syndrome, approximately 11% of bipolar risk subjects developed psychosis during a 4.5-year follow-up period.^54^ Approximately 30% of patients with an initial diagnosis of mania/bipolar disorder eventually received a different diagnosis during follow-up, and the proportion with a main diagnosis of the schizophrenia spectrum disorder increased from 4.1% at the second contact to 12.9% at the tenth contact.^55^ A recent large-scale cohort study has confirmed that there may be emergent psychotic disorders outside the CHR-P state, arising from other established ICD-10 mental disorders.^56^ It is thus possible that future research considering nonpsychotic risk syndromes could improve our ability to detect a pluripotential risk state for various mental disorders.

The second finding of our review is that prediction of new nonpsychotic disorders from an initial risk syndrome for nonpsychotic disorders is currently problematic. Our finding of a pooled incidence of 43.5 per 1000 person-years nonpsychotic outcomes in clinical risk syndromes for nonpsychotic disorder indicates that this criterion is not sufficient to predict the onset of nonpsychotic illnesses. This result may be due to the lack of specific diagnostic criteria that define clinical risk syndromes for the nonpsychotic disorder. There are currently 3 different prospective diagnostic instruments for assessing bipolar risk syndrome.^28,29,57,58^ Although these instruments are currently being used or validated, there are still few prospective studies that have used these scales, and no prospective study using these instruments has yet reported psychotic outcomes in bipolar risk syndrome. Most of the bipolar risk syndrome studies included in our systematic review were conducted with conventional clinical scales rather than with instruments specially designed to define specific risk syndromes for nonpsychotic disorder. Another fact to consider is that clinical risk syndromes for the nonpsychotic disorder may reflect relatively early-emerging phenotypes, which can contribute to low specificity of risk syndromes for nonpsychotic disorders. For example, patients with bipolar risk syndrome are more likely to have symptoms such as mood swings, sleep disorders, and poor impulse control, and these symptoms can also be observed in childhood.^59,60^ Thus, it may not be easy to distinguish true positive bipolar risk syndrome subjects from those who have mental illnesses that are common in childhood and adolescence, such as ADHD.^61,62^ Additionally, since heterogeneity of risk syndromes for nonpsychotic disorders may cause lead-time bias and a high rate of false positives, further prospective research on risk syndromes for nonpsychotic disorder should be conducted with a longer follow-up duration.

The third finding relates to the possibility of meeting CHR-P and clinical risk syndromes for the development of nonpsychotic disorders at the same time. For example, if a patient has both nonpsychotic disorder and CHR-P, it is difficult to tell whether he/she has a nonpsychotic disorder with CHR-P, or whether it is a CHR-P with comorbid nonpsychotic disorder. There are also substantial differences across CHR-P instruments, such as the Structured Interview for Psychosis-Risk Syndrome (SIPS), which considers comorbidities, and the Comprehensive Assessment of At-Risk Mental State (CAARMS), which requires a differential diagnosis with comorbid mental disorders in order to meet the CHR-P criteria.^63^ In this regard, since most studies did not implement specific instruments to detect clinical risk syndromes, there is a limit in evaluating whether they truly were risk groups or just had subthreshold symptoms. If individuals at clinical risk syndromes for nonpsychotic disorders were not evaluated by the specific instrument for CHR-P, it is hard to tell that those individuals were actually “outside” the CHR-P. Therefore, additional instruments for both psychotic/nonpsychotic risk syndromes should be employed to detect and optimize the identification of the help-seeking individuals whose future diagnosis is difficult to be predicted. Nevertheless, there are more practical alternatives to this. Recently developed individualized risk calculators help identifying individuals at-risk of developing psychosis within a nonpsychotic ICD-10 mental disorder.^56^ This may substantially reduce the effort required to repeatedly assess through multiple instruments and provide an opportunity to evaluate risk population in a more integrative manner.^64^ Our review is in line with these findings and it suggests that it is necessary to go beyond the current strategy of identifying true positives in about one-fourth of the CHR-P risk group (see supplementary material [eDiscussion] for details on factors predicting the outcomes in risk syndromes for nonpsychotic disorders).

This study has several important limitations. First, we included few articles in this systematic review, only 4 of which were prospective studies. Therefore, it is necessary to replicate our findings through further studies. Second, because most of the studies were not specifically designed to examine psychotic outcomes, the distinction between schizophrenia and mood disorder with/without psychotic features is not clear. Third, in the studies that did not report the onset of psychosis, it is difficult to determine whether the psychosis actually did not occur, or the occurrence was just not evaluated for it not being the main goal of the study. To mitigate for this problem, we included only studies that reported incident psychosis from clinical risk syndromes for nonpsychotic mental disorders. At the same time however, this may overestimate the incidence rate of psychosis in clinical risk syndromes for nonpsychotic mental disorders. Fourth, since many of the eligible studies used different rating scales/criteria, these samples may hardly be considered as unique and homogeneous high-risk groups. We hope that in the near future additional studies will be conducted using prospective designs and validated psychometric tools to define high-risk samples.

## Conclusions

Psychotic disorders may emerge outside the CHR-P paradigm, from clinical risk syndromes for incident nonpsychotic disorders, albeit at lower rates than in the CHR-P group. The clinical risk syndromes for emerging nonpsychotic disorders may exhibit a pluripotential risk of developing several types of mental disorders compared with CHR-P. If substantiated by future research, the current findings suggest that it may be useful to move beyond the current strategy of identifying individuals meeting CHR-P criteria only.

## Supplementary Material

Supplementary data are available at *Schizophrenia Bulletin* online.

eDiscussion

eIntroduction

## Funding

This research was supported by the Basic Science Research Program through the National Research Foundation of Korea (2017M3C7A1029610).
