# Supplementary material for: Can We Predict Psychosis Outside the Clinical High-Risk State? A Systematic Review of Non-Psychotic Risk Syndromes for Mental Disorders
Source: Schizophr Bull. 2018 Feb 9;44(2):276–85. doi: 10.1093/schbul/sbx173 (PMC5814842; doi:10.1093/schbul/sbx173)
Supplement: eDiscussion [file sbx173_suppl_ediscussion.doc]

**eDiscussion**

When examining the predictors of psychosis in risk syndromes for non-psychotic disorder, the presence of dysthymia and OC symptoms at baseline was highly associated with later-developing psychosis in prospective studies. In the ratio of psychotic to non-psychotic outcomes, OC risk subjects showed a considerably higher ratio than other risk syndromes for non-psychotic disorders. Since OC symptoms are often accompanied by CHR for psychosis, and they increase the risk of developing psychosis, this is not a surprising finding.^1, 2^ Although there are many reports for obsessive-compulsive symptoms as prodromal symptoms of schizophrenia, the dysthymic disorder as risk syndrome for the psychosis has not yet been thoroughly investigated. In the long-term course of illness, dysthymic disorder is known to be a diagnostically stable mental disorder; psychotic outcomes are rarely reported, although they are highly associated with major depressive disorder and bipolar disorder.^3, 4^ Therefore, additional studies are needed to determine whether dysthymic disorder is a risk factor for psychotic outcomes or whether other conditions associated with dysthymic disorder can trigger such a consequence. Although the incidence of psychosis in risk syndromes for non-psychotic disorders is considerably lower than that of CHR-P,^5, 6^ the fact that the presence of dysthymia and OC symptoms at baseline is likely to require more aggressive intervention considering the possibility of the onset of psychosis. In retrospective studies, an older age of onset of the first mania symptoms and a lower prevalence of comorbid ADHD were shown to be significant risk factors for psychosis in bipolar risk syndrome subjects. Age of onset has been a controversial issue in bipolar disorder. Earlier studies have shown that bipolar disorder began at a mean age of late twenties to thirties, but others have reported that it began as early as adolescence and that manic episodes usually manifested in the early twenties.^7, 8^ Some studies have reported that bipolar risk syndrome stage began even before the age of ten.^9, 10^ However, since the symptoms that manifest in childhood and adolescence overlap considerably with ADHD, a concern was raised that ADHD symptoms in this period were misdiagnosed as bipolar risk syndrome. Most CHR studies had enrollment criteria from 12 to 14 years of age, and all the studies showed that the mean age of the high-risk population ranged from the late teens to mid-twenties.^5^ However, the age at enrollment of psychosis risk syndrome was not a significant demographic risk factor for the onset of psychosis.^11^ Previous studies indicated that early onset of depression was the best predictor for later development of bipolar disorder.^12-14^ In a long-term follow-up study, externalizing psychopathology, insidious onset, poor premorbid adjustment, and low IQ were significant predictors for the development of psychosis in adolescents with bipolar disorder.^15, 16^ However, age of onset and patients’ age were not significant predictors.^17^ Therefore, these findings may reflect the fact that developmental disorders such as ADHD are more commonly seen at younger ages and that these disorders have a substantially lower conversion rate to psychosis.

**References**

**1.** Sterk B, Lankreijer K, Linszen DH, de Haan L. Obsessive-compulsive symptoms in first episode psychosis and in subjects at ultra high risk for developing psychosis; onset and relationship to psychotic symptoms. *Aust N Z J Psychiatry* 2011;45(5):400-406.

**2.** Fontenelle LF, Lin A, Pantelis C, Wood SJ, Nelson B, Yung AR. A longitudinal study of obsessive-compulsive disorder in individuals at ultra-high risk for psychosis. *J Psychiatr Res* 2011;45(9):1140-1145.

**3.** Kovacs M, Akiskal HS, Gatsonis C, Parrone PL. Childhood-onset dysthymic disorder. Clinical features and prospective naturalistic outcome. *Arch Gen Psychiatry* 1994;51(5):365-374.

**4.** Klein DN, Shankman SA, Rose S. Dysthymic disorder and double depression: prediction of 10-year course trajectories and outcomes. *J Psychiatr Res* 2008;42(5):408-415.

**5.** Fusar-Poli P, Bonoldi I, Yung AR, et al. Predicting psychosis: meta-analysis of transition outcomes in individuals at high clinical risk. *Arch Gen Psychiatry* 2012;69(3):220-229.

**6.** Fusar-Poli P, Cappucciati M, Rutigliano G, et al. At risk or not at risk? A meta-analysis of the prognostic accuracy of psychometric interviews for psychosis prediction. *World Psychiatry* 2015;14(3):322-332.

**7.** Weissman MM, Warner V, John K, Prusoff BA, Merikangas KR, Wickramaratne P, Gammon GD. Delusional depression and bipolar spectrum: evidence for a possible association from a family study of children. *Neuropsychopharmacology* 1988;1(4):257-264.

**8.** Fogarty F, Russell JM, Newman SC, Bland RC. Epidemiology of psychiatric disorders in Edmonton. Mania. *Acta Psychiatr Scand Suppl* 1994;376:16-23.

**9.** Kochman FJ, Hantouche EG, Ferrari P, Lancrenon S, Bayart D, Akiskal HS. Cyclothymic temperament as a prospective predictor of bipolarity and suicidality in children and adolescents with major depressive disorder. *J Affect Disord* 2005;85(1-2):181-189.

**10.** Axelson DA, Birmaher B, Strober MA, et al. Course of subthreshold bipolar disorder in youth: diagnostic progression from bipolar disorder not otherwise specified. *J Am Acad Child Adolesc Psychiatry* 2011;50(10):1001-1016 e1003.

**11.** Fusar-Poli P, Tantardini M, De Simone S, et al. Deconstructing vulnerability for psychosis: Meta-analysis of environmental risk factors for psychosis in subjects at ultra high-risk. *Eur Psychiatry* 2017;40:65-75.

**12.** Beesdo K, Hofler M, Leibenluft E, Lieb R, Bauer M, Pfennig A. Mood episodes and mood disorders: patterns of incidence and conversion in the first three decades of life. *Bipolar Disord* 2009;11(6):637-649.

**13.** Akiskal HS, Hirschfeld RM, Yerevanian BI. The relationship of personality to affective disorders. *Arch Gen Psychiatry* 1983;40(7):801-810.

**14.** Homish GG, Marshall D, Dubovsky SL, Leonard K. Predictors of later bipolar disorder in patients with subthreshold symptoms. *J Affect Disord* 2013;144(1-2):129-133.

**15.** Werry JS, McClellan JM. Predicting outcome in child and adolescent (early onset) schizophrenia and bipolar disorder. *J Am Acad Child Adolesc Psychiatry* 1992;31(1):147-150.

**16.** Ruggero CJ, Carlson GA, Kotov R, Bromet EJ. Ten-year diagnostic consistency of bipolar disorder in a first-admission sample. *Bipolar Disord* 2010;12(1):21-31.

**17.** Carlson GA. Diagnostic stability and bipolar disorder in youth. *J Am Acad Child Adolesc Psychiatry* 2011;50(12):1202-1204.
