# Supplementary material for: Can We Predict Psychosis Outside the Clinical High-Risk State? A Systematic Review of Non-Psychotic Risk Syndromes for Mental Disorders
Source: Schizophr Bull. 2018 Feb 9;44(2):276–85. doi: 10.1093/schbul/sbx173 (PMC5814842; doi:10.1093/schbul/sbx173)
Supplement: eIntroduction [file sbx173_suppl_eintroduction.doc]

**eIntroduction**

For example, a patient who later develops myocardial infarction may first experience chest pain or discomfort caused by an insufficient supply of oxygen-rich blood; this is called angina, a CHR state of myocardial infarction. Later, these symptoms may develop into a constellation of severe radiating pain, shortness of breath, sweating, nausea, vomiting and other symptoms, and the results of electrocardiography and blood chemical laboratory tests support the definite medical diagnosis of myocardial infarction. Although not all patients diagnosed with myocardial infarction have experienced the same anginal pain before the onset of the disease, early detection of angina gives patients a valuable opportunity to prevent severe, even life-threatening illnesses that may occur later.

Similar concepts of prodrome can be applied to psychiatric illnesses such as schizophrenia. Since the initial observation by Bleuler and Kraepelin, who retrospectively reconstructed the specific period in which characteristic symptoms had appeared before the onset of schizophrenia, ‘schizophrenia prodrome’ has become an important concept in explaining the course of schizophrenia. Since those early years, McGorry and Yung in Melbourne, Australia developed Bell’s “multiple-gate screening” and proposed the “close-in” strategy.^1^ In this approach, they focused on patients who were at ultra high-risk (UHR) of developing psychosis in the near future. They applied clinical criteria to differentiate these patients from past genetic high-risk subjects and set the age criteria for which the onset is considered imminent to shorten the unnecessarily long follow-up observational period. By that time, many pioneering researchers in the United States and Europe had developed unique diagnostic instruments for CHR populations or individuals with basic symptoms (BS).^2-4^ They had also established a prospective cohort program and began research for early detection and early intervention for individuals at clinical high-risk of psychosis (CHR-P). With over two decades of research, we have been able to broaden our understanding of CHR-P. The early detection of CHR-P is believed to represent an opportunity to prevent the onset of psychosis, similar to the detection of angina prior to myocardial infarction contributing to reduced mortality rates.

**References**

**1.** McGorry PD, Yung AR, Phillips LJ. The "close-in" or ultra high-risk model: a safe and effective strategy for research and clinical intervention in prepsychotic mental disorder. *Schizophr Bull* 2003;29(4):771-790.

**2.** Miller TJ, McGlashan TH, Rosen JL, Somjee L, Markovich PJ, Stein K, Woods SW. Prospective diagnosis of the initial prodrome for schizophrenia based on the Structured Interview for Prodromal Syndromes: preliminary evidence of interrater reliability and predictive validity. *Am J Psychiatry* 2002;159(5):863-865.

**3.** Cornblatt B, Lencz T, Obuchowski M. The schizophrenia prodrome: treatment and high-risk perspectives. *Schizophr Res* 2002;54(1-2):177-186.

**4.** Klosterkotter J, Hellmich M, Steinmeyer EM, Schultze-Lutter F. Diagnosing schizophrenia in the initial prodromal phase. *Arch Gen Psychiatry* 2001;58(2):158-164.

**5.** Addington J, Heinssen R. Prediction and prevention of psychosis in youth at clinical high risk. *Annu Rev Clin Psychol* 2012;8:269-289.
